# Development of the Observable Well-Being in Living With Dementia-Scale

**DOI:** 10.1177/15333175231171990

**Published:** 2023-06-02

**Authors:** Kristine G. Madsø, Nancy A. Pachana, Inger H. Nordhus

**Affiliations:** 1 NKS Olaviken Gerontopsychiatric Hospital, Askøy, Norway; 2 Department of Clinical Psychology, 1658 University of Bergen, Bergen, Norway; 3 School of Psychology, 1974 The University of Queensland, Brisbane, Australia; 4 Department of Behavioral Medicine, University of Oslo, Oslo, Norway

**Keywords:** well-being, dementia, observation, scale, psychometric properties, music therapy

## Abstract

The Observable Well-being in Living with Dementia-Scale was developed to address conceptual and methodological issues in current observational scales for music therapy. Creative interventions may receive lowered scores, as existing instruments rely heavily on verbal behavior. Methods were (1) Systematic review of observational instruments: (2) field work with music therapy and sociable interactions to operationalize the items; (3) field testing assessing feasibility and preliminary psychometric properties; (4) focus groups with experts to investigate content validity; (5) final field test and revision. 2199 OWLS-ratings were conducted in 11 participants. Hypotheses of construct validity and responsiveness were supported (r = .33 −.65). Inter-rater reliability was good (84% agreement between coders, Cohen’s Kappa = .82), and intra-rater reliability was excellent (98% agreement, Cohen’s Kappa = .98). Focus groups with 8 experts supported the relevance of the items and suggested further refinements to increase comprehensiveness. The final field-tested OWLS showed improved inter-rater reliability and usability.

## Significance statement:

• Observational instrument assessing momentary well-being during music therapy
• Outcomes assessed are clinically relevant for people living with dementia
• Preliminary results indicate promising psychometric properties and feasibility

## Introduction

Well-being is acknowledged as an important outcome for interventions by people living with dementia.^1-3^ While well-being is for the most part assessed through self-report in the general population, proxy-reports have been used in the dementia population, particularly for people living with more advanced dementia.^4,5^

Several inquiries support that people living with mild to moderately severe dementia may self-report well-being validly, even more so if facilitated through face-to face interviews, adapted response formats, and appropriate wording.^5-7^ However, increasing cognitive impairment following progressive dementia may affect responses due to memory, insight, and language deficits.^4,7,8^ Proxy-assessment and self-report using ratings of aggregated evaluations of the past weeks are especially prone to recall-bias.^9^ Additionally, consensus between proxy-assessments and self-reports of quality of life or well-being in people living with dementia is low. First, caregiver burden predicts lower proxy-ratings of quality of life for both family and professional caregivers.^5^ Second, family caregivers’ lowered well-being, health, and depression are also related to lower ratings of the care receiver.^10^ Finally, proxy-assessment systematically evaluates well-being as being lower with increasing dementia severity in a way self-reports are not.^8^ 2 important implications follow from these research findings. If the person with dementia is not able to self-report about well-being, the next best approach is to use observational measures rated by independent observers.^5^ Additionally, a large meta-analysis suggest lowered well-being and dementia severity are not systematically related,^11^ implying instruments assessing well-being should investigate the presence of such correlations. If well-being is decreasing with dementia severity, chances are the instrument is assessing other aspects than well-being. These aspects may include level of cognitive impairment through relying on verbal expressions or functional ability, both of which may be limited without necessarily adversely influencing well-being.^5,12^

For people living with dementia, retrospective self-reports may be colored by the individual’s current emotional state.^13^ A suggestion to resolve this issue is to use momentary assessments.^9^ While dementia-related symptoms may offer challenges related to negative overall evaluations of one’s life, *momentary* well-being, including happiness or enjoyment, is identified as a significant outcome.^3,6,8^ Additionally, momentary assessment has high ecological validity.^9^ It seems reasonable that these positive behavioral expressions are easier to proxy-assess through observational measures than retrospective aggregated scores of the past weeks.

A review investigating well-being from the perspective of people living with dementia, described well-being as the overall life satisfaction, which included emotional well-being, social well-being, and psychological well-being.^6^

Music therapy is promising for increasing well-being in people living with dementia,^14^ but consistent findings are lacking.^15^ Assessment of effects over longer time periods may be biased by the factors addressed earlier (i.e., cognitive, and functional declines), and momentary positive effects on well-being are more reasonable to expect due to the variety of causes of neuropsychiatric or behavioral symptoms commonly following dementia progression.^16,17^ Thus, momentary assessment may better detect these potential positive effects.

A review of observational measures assessing well-being and quality of life showed most current scales are inadequate.^18^ Particularly challenging is the lack of observational instruments assessing momentary well-being in creative interventions. A main reason is that their scores depend on *verbal* expressions,^19^ leading to lower scores for persons occupied with nonverbal activities. This is a major issue when comparing music therapy or other nonverbal interventions to more verbal interventions like cognitive stimulation therapy or reminiscence therapy. Other instruments assessing music therapy through observations include the Music in Dementia Assessment Scale,^20,21^ and Music Therapy Engagement scale for Dementia.^22^ MiDAS has a momentary focus but is not created for neutral observers. In addition, staff-ratings had low reliability.^20^ MTED gives an overall rating of a whole session without capturing smaller momentary changes essential for assessing well-being in people with more severe dementia.

A widely used instrument is the Observed Emotion Rating Scale (OERS), which has a relatively high focus on *negative* expressions.^23^ This will often lead to infrequent and skewed ratings that do not fit well with a range of statistical approaches.^24^ Algar et al.^18^ recommended the Greater Cincinnati Chapter Well-Being Observational Tool (GCWBT), but recent publications have demonstrated a lack of structural validity and low reliability in this instrument.^25,26^

Lawton’s^27^ widely cited model of well-being and quality of life in dementia suggests a two-factor model of positive and negative emotions. Negative emotions seem to be more easily modified by internal triggers, while positive emotions are more easily modified by external environment and interventions.^28^ Thus, targeting well-being through assessing positive emotions seems the most feasible approach, as negative emotions may be triggered by multiple causes, some of a chronic nature.^17^ Additionally, self-rated modifiable factors related to well-being include relationship quality, positive feelings, agency and social connectedness,^29^ guiding the domains relevant for an assessment of well-being.

Thus, the aim of this study was to develop an observational instrument to assess momentary well-being during music therapy for people living with dementia. An important objective was to develop an instrument easily adaptable to several contexts, enabling comparisons of well-being during a range of psychosocial interventions that include both verbal and nonverbal interactions.

## Method

The development of OWLS included 5 steps; 1) a literature review investigating existing observational instruments, including a review of the well-being conceptualizations in generic and dementia-specific models; 2) qualitative field work to develop operationalizations; 3) field testing of the tentative items in a clinical study (ID NCT03011723; www.clinicaltrials.gov); where reliability, construct validity and responsiveness was investigated; and 4) establishing content validity through focus groups with relevant experts that were the intended users of the scale; followed by 5) revision and a final round of field testing.

### Literature Review

The literature search was conducted in MEDLINE, EMBASE, PsycINFO, Web of Science, CINAHL, ProQuest Psychology and ProQuest Nursing and Allied Health April 21^st^ 2020 and repeated April 06^th^ 2021, using a combination of the words “well-being“, “dementia“, “observation“, “measurement“, and “psychometric properties”.^24^ Theoretical and conceptual models of momentary well-being was acquired through hand searching of publications of instruments or reviews on this topic. Qualitative research including the view of people living with dementia was consulted to ensure relevance and comprehensiveness of the items of the instrument. This led to the initial development of the items, and conceptualization of the instrument.

### Field Work

Field work was conducted with video data to elaborate the items and their operationalizations. This data came from a clinical trial (N = 11) comparing regular social interaction with music therapy.^30^

Inclusion criteria for the clinical trial were 1) ability to provide (facilitated) informed consent; 2) a formal dementia diagnosis of Alzheimer dementia, Vascular dementia, dementia of mixed etiologies or Lewy-Body Dementia (according to ICD-10 criteria52); 3) Dementia severity ranging from .5-2 on the Clinical Dementia Rating Scale55; 4) Psychotropic medications were stable at least 2 weeks before pre-assessment; 5) participants were home-dwelling or in assisted living arrangement; 6) a caregiver committing to act as collateral therapist in the study. Exclusion criteria were frontotemporal dementia, severe aphasia, severe psychosis or high risk of suicide.^30^ We decided to include people with mild to moderate dementia to optimize the potential for being able to self-report on emotional state before and after the intervention, in order to test the validity of our observations against the self-reported data. The clinical assessment of the participants with CDR and NPI-Q was conducted by 2 clinical psychologists (first author KGM & clinical psychologist Louise Markhus). Further details about the field-testing is available in Madsø et al.^30^

Utilizing the software Noldus Observer XT 12.5 ©,^31^ KGM repeatedly watched video observations of different 10-minute segments from the participants’ interactions to identify significant sections.^32^ Behavioral expressions identified as indicating well-being were described. Different sampling strategies were tested, and the codes were refined to capture the different aspects of well-being across interaction-based contexts. The items and operationalizations of the refined coding scheme were then the evaluated independently first in a team of 2 psychologists (Minna Hynninen & IHN), and secondly by an external music therapist expert (Solgunn Knardal). KGM created a coding manual and trained 2 psychology students to use the coding scheme. They gave feedback on its comprehensibility, interpretation of items, and feasibility. This process was iterative, based on recommendations for developing observational scales.^33-35^ When coders reached >80% agreement during training, the next step was conducted.

### Investigation of Psychometric Properties

**Figure 1.**
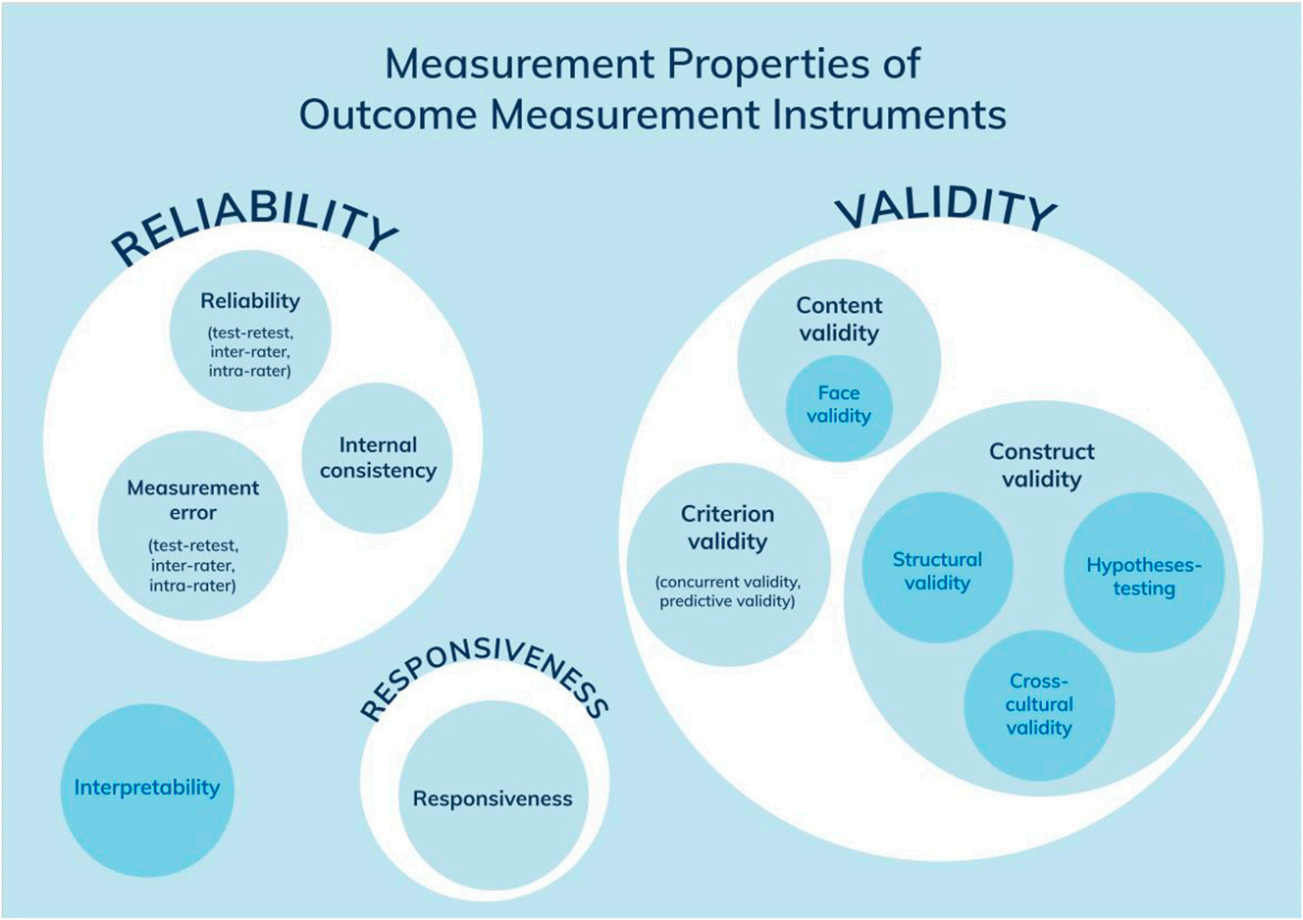
COSMIN taxonomy. Reprint of this figure from Mokkink et al. 36 is permitted under the Creative Commons Attribution 4.0 (http://creativecommons.org/licenses/by/4.0/).

To investigate psychometric properties, we used the unified terminology and definitions of the COnsensus-based Standards for the selection of health Measurement INstruments (COSMIN^36^). The COSMIN Taxonomy of Measurement Properties (Figure 1) illustrates all relevant aspects to be assessed in health-related measurement instruments.

Statistical analysis was conducted in R,^37^ and RStudio,^38^ and reliability measures were provided by the Noldus Observer XT © software.^31^

#### Reliability and Agreement

Approximately 20% of the total material was coded by both a research assistant and the main coder (KGM). Inter-rater reliability was assessed with Cohen’s Kappa, which is a relative measure of instrument reliability.^39^ Inter-rater agreement was assessed as % of agreement, as recommended for dichotomous instruments.^40^ Percentage of agreement is an absolute measure, indicating measurement error of nominal levelled instruments.^39^

#### Construct Validity

As no gold standard instrument was available for comparison, criterion validity could not be assessed.^36^ Thus, construct validity was assessed through correlations with a similar instrument from the clinical study.^41,42^ The Verbal and Nonverbal Interaction Scale – Care Recipient (VNVIS-CR^43^) assesses momentary sociable and unsociable verbal and nonverbal behavior towards significant others and consists of a total scale and 2 subscales: sociable-verbal and sociable-nonverbal interaction. VNVIS-CR has adequate psychometric properties (inter-coder reliability .92, test-retest reliability r = .61-.77, internal consistency (α = .65-.79), testing of construct validity found significant correlations with hypothesized scales or items of scales measuring positive and negative emotions (r = .59-73) and depression measure (r = −.34), and predictive validity). The VNVIS-CR sum scores was correlated with OWLS sum scores. We expected the total scale to co-vary with the well-being scale. As we aimed to investigate nonverbal behavior specifically, we expected a higher correlation with the nonverbal subscale to support the ability of our instrument to detect nonverbal behavior. The following a priori hypotheses were formulated based on the generic hypotheses of Prinsen et al.,^41^ and investigated through Pearson’s correlations:

I. VNVIS-CR subscale Nonverbal Interaction will correlate higher with OWLS than the total VNVIS-CR
II. OWLS will correlate between .30 - .70 with VNVIS-CR subscale Nonverbal Interaction

#### Responsiveness

Responsiveness is *“the ability of an* [instrument] *to detect change over time in the construct to be measured*”^36^(p 743). Responsiveness was investigated through correlations with the change-scores of instruments measuring similar constructs.^41,42^ Effect-sizes (Log Response Ratio (LRR))^44,45^ calculated from each single-case intervention in our clinical study was expected to correlate with the change-scores calculated from pre-to post session on the self-reported subscale “happy” in the Visual Analogue Mood Scale (VAMS),^46^ and the change scores from pre-to post-total intervention period (10 weeks) of the Neuropsychiatric Inventory-Questionnaire (NPI-Q).^47^ A priori hypotheses postulated by the research team were based on the generic hypotheses of Prinsen et al.^41^:

I. OWLS effect size (LRR) will correlate ≥ .30 with the change score from pre-to post session with VAMS-item “happy”
II. OWLS effect size (LRR) will correlate ≥ .30 with the change score from pre-to post intervention from NPI-Q

The first author who also coded video-data, was blind to the hypotheses of construct validity and responsiveness.

### Expert Assessment

To assess content validity of the field-tested version of the instrument, we conducted 2 focus groups. We developed a semi-structured interview-guide.^48^ The interview guide included open questions about the understanding of relevant experts of what well-being was in general, and specificly for people living with dementia. The interview guide was based on criteria from Terwee et al. (Table 4, p 1166).^49^ Next, the relevant aspects of content validity of the health-related instruments were assessed, including the relevance, comprehensiveness, and comprehensibility of the instrument.^49^ The current version of OWLS was provided to discuss in the focus group the comprehensibility of the instructions, wording, and scoring of the instrument. Relevance and comprehensiveness were assessed for the construct of well-being, the target population, and the context of use. Context was defined as the psychosocial interventions the experts were familiar with and used in their daily work with people living with dementia. Lastly, the participants were asked if all key concepts were covered.

Informed consent was provided, and interviews were recorded and transcribed verbatim. Analysis followed a selective coding procedure^48^; as it was conducted to refine the items already identified during literature review and field testing.^35^ We looked for keywords close to the spoken descriptions from the experts, as well as examples describing the different aspects of the topic.^50^

### Revision and Final Field Test

The suggestions from the focus groups were evaluated during a final field test including 10% of the observations from participants from the initial field study. This led to the final revision of the instrument. KGM assessed of intra-rater reliability and agreement in this field test by coding 10% of the material twice, with a one-week interval.

Finally, we compared the items in the final version of OWLS to theoretical models of well-being and qualitative inquiries about important outcomes for people living with dementia, to validate the conceptualization-model of momentary well-being on which our instrument is based.^33,34^

### Ethics

The participants in the clinical study gave written informed consent, and ethical approval was provided by the Regional Committees for Medical and Health Research Ethics in Norway (2016/1374). The clinical trial was pre-registered at www.clinicaltrials.gov (ID: NCT03011723). For the focus groups, data handling was approved by the Norwegian Centre for Research Data (ID: 489856), and all participants gave written informed consent.

## Results

### Literature Review

The work of Clark et al^6^ guided the conceptual work of the team, which describes 6 domains capturing the lived experiences of well-being in people with dementia; “Feeling positive”, “Live having meaning”, “Positive sense of self”, “Keeping going and being active”, “Good relationships” and “Feeling well”. Other conceptualizations of well-being in dementia were also consulted.^27,53,54^ The literature search identified 22 different instruments assessing momentary well-being through observation. Content validity is always context dependent,^49^ and none of the identified instruments demonstrated feasibility in regard to the research questions addressed in our clinical study. However, item-operationalizations close to our aims were found in the instruments Observed Emotion Rating Scale,^23^ Observable Displays of Affect,^19^ the Greater Cincinnati Chapter Well-Being Observational Tool,^51^ and Music in Dementia Assessment Scales.^20^

### Field Testing of the Instrument

The observational material provided from the clinical study consisted of 11 people living with dementia, observed on 6 different occasions. The participants were aged 71 – 88 years (m = 79.82, SD = 5.27), 63% were women, and the clinical dementia stage ranged from mild to moderately severe. Dementia diagnosis according to the ICD-10^52^ were Alzheimer’s dementia (9) and Vascular Dementia (2). All participants were living at home. All observations included interactions with a family caregiver and a music therapist. To decrease signs of reactivity to the video-camera the music therapist explained the rationale for using video-recording every recorded session. The participants were given time to get used to the camera and to consent to this procedure each time if the participant had forgotten about the recording.

Examples of 2 significant sections are provided in Table 1.

**Table 1.** Qualitative descriptions from the field work.

Example of “Identity” and “Mastery”
Context: The music therapist is sitting in a chair, with participant “Iris” sitting in a sofa and the husband sitting in another chair. The music therapist offers “Iris” to hold and maybe play the guitar. “Iris” responds that she might try and reaches out for the guitar. She describes which chords she used the most, and they play a song together.
Significant section:
“Iris”: [stretching her fingers and sighs]. My fingers hurt; I really cannot play the guitar now days. [Face express sadness, looks down, shoulders sunken].
Therapist: [soft voice] Oh, but I can see you know how to play. [gains eye contact].
“Iris”: Oh, I used to be very good at playing the guitar, you know! [face lights up in a smile, she straightens up her body and looks at the music therapist and then at her husband]
Husband: [Nods and smiles at “Iris”]
Example of “relationship”
Context: The music therapist is sitting in a chair, with “Beth” and her daughter sitting in a sofa. “Beth” has been quiet for some time, and the music therapist have asked if they should play a song together.
Daughter: [Holds her mother’s hand while sitting next to her]
“Beth”: [Absent gaze; looking into the room without focusing on anything. Body is still]
Therapist: [Plays guitar and sings the chorus of ABBA’s *“Dancing queen”,* a song she knows “Beth” is familiar with] …Oh-oh, see that girl, watch that scene, digging the …[pauses in the song and leans towards “Beth”].
“Beth”: [Turns her head towards the music therapist. Suddenly a lively gaze in the eyes. Sings along the last word of the phrase after a 3 second break] …Dancing queen. [Laughs while turning her head towards her daughter and looks at her.]
Daughter: [Smiles while turned towards mother, still holding her hand]. “You like that song, don’t you mom?”
“Beth”: [Laughs and has eye contact with daughter for a few seconds. Turns head and return to absent gaze]

Thus, based on the most appropriate and well-cited theoretical conceptualizations of well-being in dementia,^6,27,53,54^ the literature review of scales described above, the qualitative observations and descriptions of well-being themes, and consultation within our team and with an experienced music therapist, 10 items were drafted. These were “attention”, “initiative/response”, “happiness”, “joking”, “enjoyment”, “mastery”, “self-confidence”, “reminiscence”, “positive feedback” and “relationship”.

**Figure 2.**
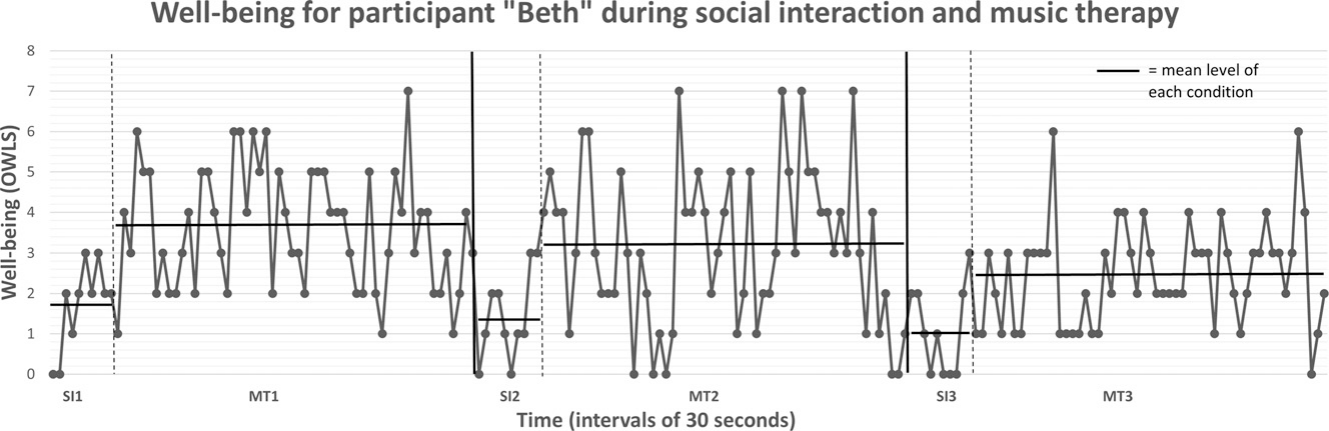
Graphical presentation of OWLS Note: Each point represents the sum of OWLS for the current 30-second interval. SI = observations during social interaction. MT = observations during music therapy. The horizontal line represents the mean level of the specified phase, enabling comparison.

After field-testing different coding approaches, interval recordings with dichotomous scoring were chosen. After observing 30 second intervals, any presence of an item-indicator led to a score of “1”, and absence led to “0”. For each interval, the presence of items is summarized, enabling a graphical presentation of the estimated well-being level as it unfolds over time (see Figure 2 for an example). In total, 2199 ratings of the total scale were included in the psychometric evaluations; 320 observations during regular social interaction and 1879 during music therapy.

### Psychometric Properties

Results from testing of reliability and measurement error are presented in Table 2. Inter-rater and intra-rater reliability and agreement were strong (κ ≥ .70, agreement ≥70%).^40^ The hypotheses about construct validity and responsiveness were supported. In addition, an exploratory analysis investigating correlations with dementia severity (Clinical Dementia Rating (CDR)^55^ showed that an increase in the change-score of well-being increased with dementia severity. This suggests the instrument can detect high scores of well-being even with increasing cogntitive impairment.

**Table 2.** Reliability, construct validity and responsiveness.

	N_o_	Score	*P*-value	Range
Inter-rater reliability				
Inter-rater agreement	417	84% agreement	—	77 – 88%
Cohen’s Kappa	417	K = .82	<.0001***	.72 – .89
Intra-rater reliability				
Intra-rater agreement	220	98% agreement	—	94 – 100%
Cohen’s Kappa	220	K = .98	<.0001***	.94 – 1
Construct validity				
Scale	N_o_	Pearson’s correlation with OWLS	*P*-value	CI
VNVIS-CR total ratio	2199	.37***	<.001***	.34 – 1.00
VNVIS-CR nonverbal ratio	2199	.65***	<.001***	.64 – 1.00
Dementia severity (CDR)^a^	32	.56***	<.001***	.26 – .76
Responsiveness				
Scale	N_o_	Pearson’s correlation with OWLS	*P*-value	CI
NPI-Q total change	32	.42	.017*	.08 – .67
VAMS ‘happy’ change	32	.33	.037*	1.3 – 1.00

Abbreviations: N_o_ = number of observational assessments per scale included in analysis, nested in the 11 participants. VNVIS-CR = Verbal and Nonverbal Interaction Scale - Care Receiver, ranging from 0-13 each assessed time-interval. CDR = Clinical Dementia Rating, higher score reflects more severe dementia. NPI-Q = Neuropsychiatric Inventory-Questionnaire, higher scores reflect more change (lower symptoms) from pre-to post intervention. VAMS = Visual Analogue Mood Scale- Items range from 0-100, where a higher score reflects more change (increased happiness) from pre-to post session.

^a^Clinical Dementia Rating was included as results from the intervention study showed dementia severity predicted increased effect of the intervention.

### Qualitative Analysis of Focus Groups

Eight experts discussed content validity of the instrument in 2 focus groups. Group 1 was assessing the instrument for use in different short time psychosocial interactions and interventions (N = 4; psychiatrist, psychologist, nurse, and social educator). Group 2 was assessing the instrument for use in music therapy (N = 4 music therapists). The experts had a mean experience of 8 years (SD = 5.5) of working with people living with dementia. The focus groups gave valuable advice about instructions and scoring to increase the comprehensibility of the instrument. They suggested that micro-expressions/behaviors should be incorporated into OWLS to improve the feasibility of the instrument in people with more severe dementia. Both focus groups stressed the complexity and necessity for interpreting signs of well-being when dementia severity increased. Including idiographic expressions of well-being, as well as familiarity with the person’s life and personal history, was emphasized. Well-being in dementia was described as including 2 main domains 1) personal emotional experiences and 2) interactional elements. The words to describe caregivers were changed to “significant other”, enabling any significant person to be the target for interaction during interventions.

Regarding comprehensiveness, 3 additional items were suggested: calm/relaxed, significant emotional experiences, and participation. Both groups highlighted how they observed facial and bodily tension, volume and tone of voice and pace of breath to evaluate presence of well-being or ill-being in people living with dementia. Reminiscence was rephrased as “express identity”. The descriptive themes under the suggested item “significant emotional experiences”, were incorporated under the item “express identity”. Both groups identified that the emotional experiences of significance in psychosocial interventions could include tearfulness, for example. Still, it was not regarded as ill-being when the emotional expression included processing of something meaningful and relevant for the individual. “Joking” was redefined to exclude making fun of others in a negative way.

All items except “self-esteem/acceptance” were regarded as relevant. We removed this item, as it was not interpreted as a momentary state and was complex to assess through observation. Key words and examples from the focus groups were used in the descriptions of the items. Items suggested based on the focus groups’ feedback were “participation”, “attention”, “initiative/response”, “calm/relaxed”, “happiness”, “joking”, “enjoyment”, “express identity”, “mastery”, “positive feedback” and “relationship”.

### Final Field Testing

The final field test included 5-minute video segments from all participants. KGM coded 10% of the video-material with the revised instrument. Re-coding of the material was conducted after a one-week interval, to assess intra-rater reliability. The item “participation” was removed, as it was anticipated to be covered well under “attention” and “initiative/response”. Additionally, we were worried this item would tap functional impairment and over-estimate well-being in people not able to leave the interaction voluntarily. “Calm/relaxed” worked well with the operationalization identical to this item in the VNVIS-CR^43^ from the initial field test. “Joking” was only coded as well-being when humor was used to strengthen the social bonds in the interaction, and we decided to incorporate “joking” under “relationship”. The analysis of intra-rater reliability uncovered that “positive feedback” was problematic to separate from “relationship”, and these 2 items were merged. This led to slight increases in intra-rater reliability (mean Kappa .95 to .98) and intra-rater agreement (from 96% to 98%). Thus, the final OWLS instrument included 8 items: “attention”, “initiative/response”, “calm/relaxed”, “happiness”, “enjoyment”, “express identity”, “mastery”, and “relationship”. The current version of OWLS is presented in the appendix.

### Interpretation and Clinical Utility

The 8 items in OWLS and their related operationalization were developed to detect observable dementia-specific well-being, and to optimize the clinical utility of the instrument. All items have both verbal and nonverbal indicators, making the scale independent of verbal expressions.

The order of the items reflects an increasing complexity of the observed expressions. Some scalability is implied for the first 4 items. “Attention” comes first, followed by “initiatives or responses”. If both these are present, “calm and relaxed” may be scored as a response to the interaction. Next, “happiness” is scored when smiling or laughing is present. The final 4 items (enjoyment, express identity, mastery and relationship) may occur alone or simultaneously, but all of them require the presence of the first 4 items. For example, “enjoyment” cannot be scored unless the first 4 states are present (see scoring-instructions). This was decided by the research team during the first field-test, to ensure that, for example, “express identity” or other items are 1) related to the activity the participant is engaging in (because they show “attention” towards the interaction and contributes with an “initiative or response”), and 2) the behavioral expression is related to a positive state in the person (because they are “calm and relaxed” and express “happiness”). The difference between “happiness” and “enjoyment” is the expressions of an increased level of absorption in the activity in the “enjoyment” coding. This is elaborated in the description of the item in the scale. While happiness may be indicated by the mere expression of a smile or expressing positive feelings, enjoyment is coded when the person signals introverted or extroverted pleasure or extroverted engagement with the activity.

The total score ranges from 0 – 8, where higher scores indicate greater well-being. Scores < 2 indicate lack of attention towards or participation in the activity or interaction in the current observational interval. Scores from 3 – 4 reflect a relaxed and positive state, a score of 5 represents a moderate intensity of well-being, and scores from 6 – 8 represent a high level of well-being.

A sum of the presence of items for each 30-second interval is calculated, enabling a graphical presentation of well-being over time. An example from the field study is presented in Figure 2. Furthermore, the relative frequency of each item may be summarized for the total intervention period, giving an estimate of the intensity of this specific item during the interaction. Examples of this are shown in Table 3. This table is based on data from the field-study.^30^ The relative frequency is calculated as number of intervals the items is present, divided by the number of intervals of observation in total.

**Table 3.** Items and corresponding frequency of OWLS in 2 contexts.

Item	Music Therapy, %	Social Interaction, %
Attention	98.5	92.8
Initiative/response	97.4	95.0
Calm/relaxed [new]	91.5	70.6
Happiness	49.8	26.6
Enjoyment	48.7	2.8
Express identity	44.7	8.2
Mastery	9.9	2.5
Relationship	45.6	11.3
Positive feedback [merged with relationship]	21.7	7.6
Joking [merged with relationship]	18.3	18.6
Self-confidence [removed]	6.2	4.7

Note: The frequencies provided in this table origin from the clinical study^30^ and may ease interpretation of scores in other similar contexts including people with mild to moderately severe dementia.

## Discussion

In the current paper, we presented the development of OWLS. The instrument assesses observable momentary expressions of well-being in people living with dementia during social interaction and music therapy. The items were constructed based on general and dementia-specific theories of well-being, an extensive literature review of existing observational instruments measuring well-being, and an iterative process of repeated field testing, assessment of psychometric properties, and focus-groups with professional experts.

The content validity of OWLS is supported by literature and theory regarding well-being in dementia.^6,27,53,54^ as well as inquiries about self-reported domains important for people living with dementia.^1-3,29^

 With increasing dementia severity, attention may fluctuate.^56^ Thus; observing “attention” is required to ensure the participant is focused on the current activity. OWLS requires the rater to first establish the direction of the observed expressions to make any inferences about well-being. Next, “initiative or response” indicates active participation, emphasized by the experts as an important indicator of establishing the potential for well-being in people with more severe dementia. As apathy is common with increasing dementia severity,^17^ observing “initiative or response” is required for establishing the person’s interest in the current activity. Then, “calm and relaxed” constitute the first level of well-being, signaling safety^2^ and comfort (Kitwood, 1997). “Happiness” and “enjoyment” are described as essential aspects when people living with dementia evaluate their quality of life,^8,29^ and as core outcomes of interventions.^2^ “Express identity” and “mastery” are observable expressions related to central inner experiences of well-being theory in dementia, encompassing maintaining personhood and identity,^54^ as well as agency.^6,29,53^ These reflect central important outcomes reported by people living with dementia as well, such as sense of competence,^3^ and keeping one’s own identity.^2^ Finally, the social aspect of well-being,^6^ is encompassed by “relationship”, reflecting the transactional behavior of maintaining close relations,^2^ attachment,^54^ and participating in social interactions with significant others.^3,29^

The anchoring of our items in well-being theory and former qualitative inquiries including people living with dementia helps to ensure that the 8 items reflect relevant and comprehensive aspects of well-being. This is further supported by the preliminary psychometric assessment of construct validity and responsiveness. Additionally, results indicate that inter-rater agreement is good and intra-rater agreement is excellent in OWLS, demonstrating reliability of the outcome scores for research settings.

The field test demonstrated the feasibility of OWLS for observing music therapy and regular social interactions with family caregivers. OWLS may prove useful for a wide range of health professionals applying observational methods. The strong focus of using nonverbal *and* verbal indicators for each item makes the instrument feasible for people with more severe dementia, as well as for comparing verbal and nonverbal interventions. The instrument captures domains that may be relevant for other creative or interactional interventions as well, and we believe it is appliable in for example art therapy, animal assisted therapy/pet therapy, horticultural or garden therapy, and reminiscence therapy. The momentary nature of the measure offers possibilities of comparing different interactional interventions in dementia on a multidisciplinary level, which is of value when choosing and tailoring interventions to individual needs.

Training of coders until an inter-rater agreement over 80% with the main coder was reached after approximately 2 days of practice. This training included education about dementia. The focus group discussed whether we should include idiographic expressions of well-being, and this is an option in the final version of OWLS. This also requires the observer to know the person they are assessing. This is specifically relevant for the items “express identity”, or when tearfulness is interpreted as processing something meaningful for the individual. The trained observers in the field-test did not know the participants, but still they reached an inter-rater agreement over 80%. As the example in Table 1 shows, the expression “Oh, I used to be very good at playing the guitar, you know” could be interpreted as expressing identity even by neutral observers. We believe OWLS can be used by both neutral and familiar observers, but including idiographic expressions requires more familiarity with the participants.

The instrument is feasible for video-recordings, and supportive software is an advantage but not a requirement. Use in direct observation was not tested in our field-work but would probably require 30 second observations followed by 30 seconds of coding similar to other instruments relying on live interval-recording.^57,58^

OWLS items measure well-being dichotomously on a nominal level. The opportunity to calculate item-specific frequency during an intervention allows for comparing content in different interventions. This enables clinicians or researchers to compare interventions through Chi-square analysis, as suggested in other observational studies.^58,59^ These frequencies will ease interpretation of the clinical relevance of item specific change in level during different interventions.

The total-score for each interval-recording enables the level of well-being to be plotted graphically. We infer some scalability, where higher scores are interpreted as higher levels of well-being. However, as momentary well-being levels seem to fluctuate due to the nature of dementia, mean levels of well-being during the specific intervention may give a better estimate of well-being level than the scores per interval. In our field-test we used a single-case calculator^60^ to compare the differences between 2 contexts, with the effect-size LRR as output.^45^ The LRR is easily recalculated to % of change between the compared conditions (i.e. baseline and intervention), which increases interpretability. During our field-test, we learned that OWLS can detect statistically significant changes. We suggest investigating change using LRR, and interpretations may be guided by benchmarks defining clinically relevant changes. We interpret <20% as no change, 20-50% as a small change, 50-70% as a moderate change, and >70% as a large change.^30^

Required training to use OWLS is to have basic knowledge about dementia, become familiar with the manual and coding instructions, and assess intra-rater reliability for the individual coder using the scale. If several coders are using OWLS, inter-rater percentage of agreement should be assessed. Reliability should reach ≥ 80% of agreement.^40^ The coding is also described in such a way that knowing the client personally is not required. Theoretically, the instructions are simple enough that a variety of health care professionals could use the OWLS in research, but it is not designed to be used by professional carers or family members. OWLS could also be used clinically to provide evidence of change over time with respect to progress of a clinical intervention, but this would need to be evaluated in a further study.

### Limitations

Content validity was our main concern when developing OWLS, as this is the most important measurement quality of any health-related instrument.^49^ Development of observational instruments is an iterative process, and the focus groups were conducted after the initial development, field-testing, and assessment of psychometric properties. Thus, the final version that was field-tested and revised has the strongest evidence of content validity but has not yet been thoroughly tested for evidence of other measurement properties.

Ideally, the focus groups would have included experts from the target population as well as the professional experts,^49^ but due to the COVID-19 pandemic we could not include people living with dementia or their family caregivers. We sought to compensate for this by assessing other qualitative literature investigating well-being from the perspective of people living with dementia. Focus groups adressing the relevance of the items capturing the voice of people living with dementia is also an area for future research.

While a high number of observations were conducted for the initial field testing, the observations were not independent but originate from a small sample size. We could not correct for the dependent observations. Consequently, the statistical analyses of construct validity and responsiveness are preliminary and warrant further investigation in larger samples. The correlation of OWLS with self-rated happiness was in the hypothesized range of .3-.7, but still it was quite small. However, responsiveness is calculated using 2 or more *change scores* and are naturally more prone to measurement error. Consequently, smaller correlations are expected than for correlations of construct validity using *single scores*.^39^

The increasing LRR effect size of OWLS correlating with increasing dementia severity is merely explorative and uncertain. Still, it provides preliminary evidence that the well-being scores of OWLS are not decreasing with increasing dementia severity as many other instruments do.

Reliability was good when tested with 3 different coders. Still, item-specific inter-rater agreement and measurement error is preferrable for item-specific analyses and is recommended in future studies utilizing this approach. If the total scores are treated as continuous in statistical parametric analyses, a better alternative to Cohen’s Kappa is to calculate intra class correlation with a two-way random effects model of absolute agreement (section 5.4).^39^

### Conclusion and Future Recommendations

We believe OWLS is a promising instrument for assessing the process of well-being during music therapy, solving issues present in other existing observational instruments^24^ OWLS is created to be an easily adaptable instrument for a variety of psychosocial interactional interventions. In future studies we recommend assessing construct validity through correlations with adequate self-reported instruments measuring momentary well-being,^6,61^ in larger samples and other relevant contexts.

## Supplemental Material

Supplemental Material - Development of the Observable Well-Being in Living With Dementia-Scale Click here for additional data file. Supplemental Material for Development of the Observable Well-Being in Living With Dementia-Scale by Kristine G. Madsø, Nancy A. Pachana, and Inger H. Nordhus in American Journal of Alzheimer's Disease & Other DementiasÂ®.

## Appendix

### List of abbreviations:

CDR: Clinical dementia rating
COSMIN: Consensus based Standards for selection of health Measurement

### Instruments

GCWBT: Greater Cincinnati Chapter Well-Being Observational Tool
ICD-10: International Classification of Diseases 10th Revision
LRR: Log Response Ratio
MiDAS: Music in Dementia Assessment Scales
MTED: Music Therapy Engagement Scale
NPI-Q: Neuropsychiatric Inventory-Questionnaire
OERS: Observed Emotion Rating Scale
OWLS: Observed Well-being in Living with dementia Scale
VAMS: Visual Analogue Mood Scale
VNVIS-CR: Verbal and Nonverbal Sociable Interaction Scale- Care Receiver
