# Supplementary material for: Development of the Observable Well-Being in Living With Dementia-Scale
Source: Am J Alzheimers Dis Other Demen. 2023 Jun 2;38:15333175231171990. doi: 10.1177/15333175231171990 (PMC10624086; doi:10.1177/15333175231171990)
Supplement: Supplemental Material - Development of the Observable Well-Being in Living With Dementia-Scale [file sj-pdf-1-aja-10.1177_15333175231171990.pdf]

Appendix 1: Observable Well-being in Living with Dementia-Scale (OWLS)

This instrument is used to code observed expressions of well-being in people living with dementia when participating in activities that include social interaction or music therapy. Video recordings of the observation period are used to increase the accuracy of coding. Knowing some background information about the person living with dementia is of value for scoring expressions reflecting the activity is personal meaningful.

Scoring: The person is observed for 30 seconds. All items are scored dichotomously as “1” if “present” and “0” if not present. To score “1”, one or more indicators of the item must be present during the 30-second interval. All the items include both verbal *and* nonverbal signs of well-being. Thus, any item may be scored as “present” when only nonverbal indicators are present. If item 1 attention and 2 initiative/response is not scored as present, none of the other items is scored in the 30-second interval. The first 4 items are required to be present before item 5, 6, 7 and 8 may be scored. Item 5-8 can be scored individually of each other or co-occur. This scalability is illustrated in the figure below.

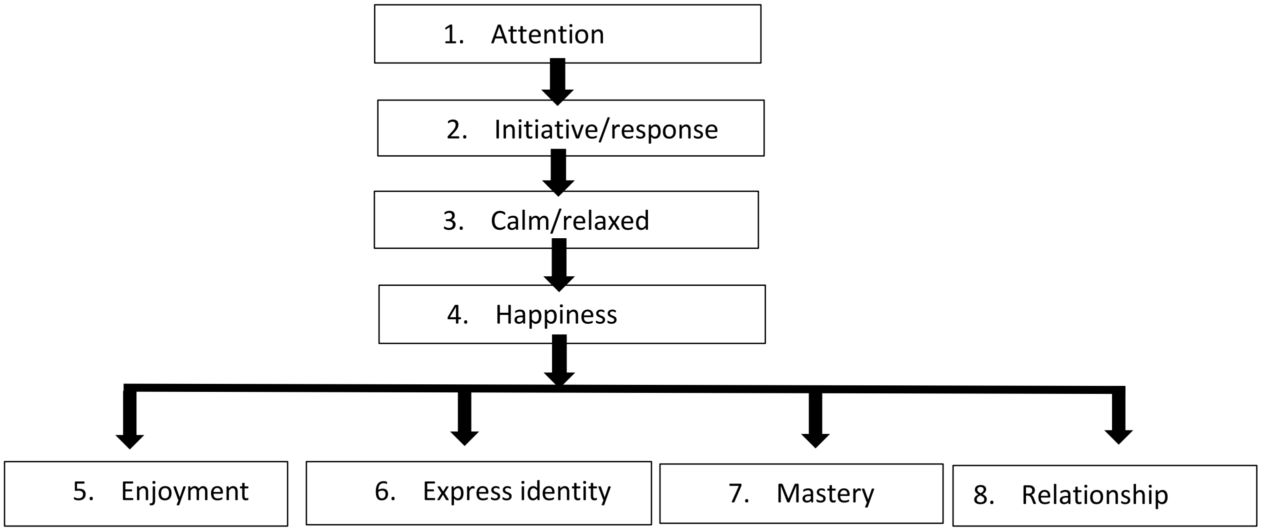

For example, MARY is observed. She is paying attention, has initiated a response, is relaxed (not tense) in the moment, and is happy. In that moment she shares her feelings with her partner (indicating relationship) about enjoying the music – for that interval her score is recorded as 6, as illustrated below.

OWLS scoring sheet:

Item		Description	Ex.	1	2	3	4	5	6	7	8	9	10	Sum
1	Attention	**Attention towards the shared interaction during the interval**	1											
2	Initiative/ response	**Responds to the initiatives of others or makes own initiatives during the shared interaction or activity**	1											
3	Calm/ relaxed	**The person is calm and relaxed during the interaction/activity**	1											
4	Happiness	**Expressions of happiness, enthusiasm, or any other positive affect**	1											
5	Enjoyment	**Signals enjoyment towards activity or interaction.**	1											
6	Express identity	**Initiative or response during activities and interactions that are related to their sense of self**	0											
7	Mastery	**Expressions of mastery in the current situation**	0											
8	Relationship	**Seek to participate in a relationship**	1											
		Sum	6											

Interpretation: The sum of each observational interval (vertical column) is used to indicate the level of well-being in this interval. If the sum is equal to or under 2, this indicates lack of well-being as the person was not attentive nor participated during the interval. A score above 2 indicates that the potential of well-being is met, and higher scores are indicative of a higher level of well-being. Scores of 3 – 4 indicate a positive state. 5 reflects moderate well-being intensity, and scores from 6-8 indicate high levels of well-being.

The sum of each item during the intervention period (horizontal row) may be calculated to estimate the intensity/magnitude of each item during the intervention period. A relative frequency is calculated by dividing the sum of the items with the number of observational intervals.

For comparing different interventions, the effect size Log Response Ratio may be calculated (https://jepusto.shinyapps.io/SCD-effect-sizes/; Pustejovsky & Swan, 2018). Changes below 20 % is regarded as no change, 20 – 50% is regarded as a small change, 50 – 70% is regarded as a moderate change, and changes above 70% is regarded as large.

	Elaboration of each item and the corresponding operationalization	
1	**Attention**	**Attention towards the shared interaction during the interval.** Displayed as gaze or bodily position facing towards interaction or activity with a clear intention. Even if the persons attention is fluctuating, this item is scored if observations of contact is present during the interval
2	**Initiative/ response**	**Responds to the initiatives of others or make own initiatives during the shared interaction or activity.** Look for body language, facial displays, and bodily positions. In more severe dementia this may be displayed in any micro-responses (reach out hands, point at, turn towards. I.e., pedal the pace during music)
3	**Calm/ relaxed**	**The person is calm and relaxed during the interaction/activity.** Comfortable posture, relaxed facial expression, shoulders relaxed, breath is calm, volume and tone of voice is neutral (not raised voice), bodily tension is reduced or not present.
4	**Happiness**	**Expressions of happiness, enthusiasm, or any other positive affect.** Smiles or laughs when attentive to activity. Simple short “social smiles” are not sufficient to score as “present”
5	**Enjoyment**	**Signals enjoyment towards activity or interaction.** May be introverted (signaling pleasure with closed eyes, relaxed bodily position, smooth facial muscles, in combination with attention towards activity or interaction) or extroverted (such as energetic, engaged gestures and actively changing bodily positions in order to respond to interaction or activity)
6	**Express identity**	**Initiative or response during activities and interactions related to their sense of self** Initiative: the person is initiating talking or doing an activity related to their identity and personal history. Response: Clear recognition in the person when others initiate interaction with themes or activities related to their identity and personal story. Positive bodily response showing the topic is of personal meaning. These themes may bring about positive emotions, but tearfulness or other emotions showing the topic is significant may as well be present. The key is for the activity to bring about meaningful and significant associations and memories related to the identity of the person, their life and experiences, and safety to express any emotion connected to this interaction
7	**Mastery**	**Expressions of mastery in current situation** Face, body, gestures or word express pride and mastery. The person communicates something positive or significant about own current or previous activity or achievement
8	**Relationship**	**Seek to participate in a relationship** Nodding or showing supportive nonverbal and verbal feedback towards significant others. Initiating to create or maintain a relationship or closeness with significant others during activity or interaction. Turn-taking interactions. Inviting significant others to share or exchange experiences. In people with more severe dementia, observing micro behavior such as gaze towards, bodily position towards, lean towards, and/or reach out to significant others. Using humorous language or gestures that is not exploiting others may be coded here as well.

Note: Idiographic signs of well-being or intervention-specific examples and descriptions may be added under the different items. Coder-specific inter-rater agreement and reliability ought to be assessed.
